# A Comparative Study on Two Typical Schemes for Securing Spatial-Temporal Top-*k* Queries in Two-Tiered Mobile Wireless Sensor Networks

**DOI:** 10.3390/s18030871

**Published:** 2018-03-15

**Authors:** Xingpo Ma, Xingjian Liu, Junbin Liang, Yin Li, Ran Li, Wenpeng Ma, Chuanda Qi

**Affiliations:** 1 School of Computer and Information Technology, Xinyang Normal University, Xinyang 464000, China; yunfeiyangli@gmail.com (Y.L.); liran@xynu.edu.cn (R.L.); markwinpe@gmail.com (W.M.); qichuanda@sina.com (C.Q.); 2 Henan Xinyang Senior High School, Xinyang 464000, China; XingjianLiu@gmail.com; 3 School of Computer and Electronic Information, Guangxi University, Nanning 530004, China; liangjb@gxu.edu.cn

**Keywords:** mobile edge computing, two-tiered mobile wireless sensor networks, top-k query, integrity preservation

## Abstract

A novel network paradigm of mobile edge computing, namely TMWSNs (two-tiered mobile wireless sensor networks), has just been proposed by researchers in recent years for its high scalability and robustness. However, only a few works have considered the security of TMWSNs. In fact, the storage nodes, which are located at the upper layer of TMWSNs, are prone to being attacked by the adversaries because they play a key role in bridging both the sensor nodes and the sink, which may lead to the disclosure of all data stored on them as well as some other potentially devastating results. In this paper, we make a comparative study on two typical schemes, EVTopk and VTMSN, which have been proposed recently for securing Top-*k* queries in TMWSNs, through both theoretical analysis and extensive simulations, aiming at finding out their disadvantages and advancements. We find that both schemes unsatisfactorily raise communication costs. Specifically, the extra communication cost brought about by transmitting the proof information uses up more than 40% of the total communication cost between the sensor nodes and the storage nodes, and 80% of that between the storage nodes and the sink. We discuss the corresponding reasons and present our suggestions, hoping that it will inspire the researchers researching this subject.

## 1. Introduction

As the Internet of Things [1,2,3,4] and the upcoming 5G communications [5] quickly develops, a paradigm shift is occurring from centralized mobile cloud computing [6] toward mobile edge computing (MEC) [7]. The main feature of MEC is to push network control, data storage, and mobile computing to network edges (e.g., access points and/or base stations) so that many latency-critical and computation-intensive applications are enabled at resource-limited mobile devices [7].

**Figure 1:**
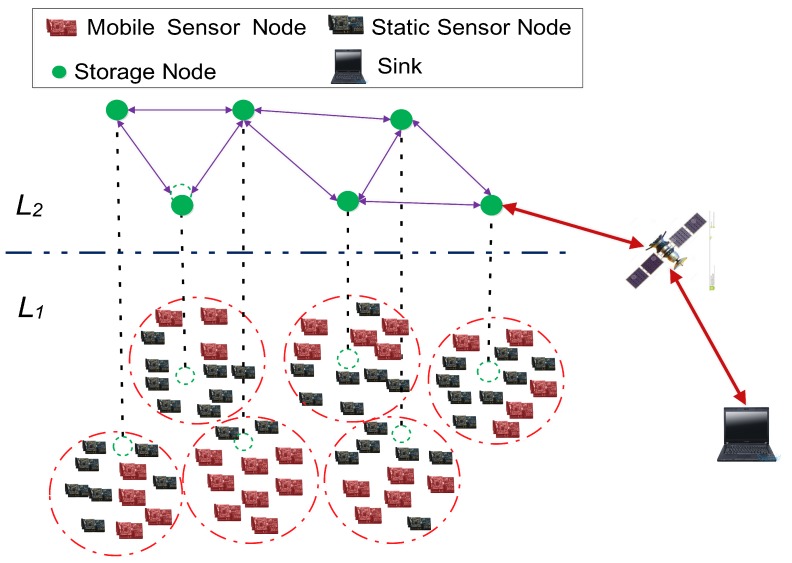
The network model of two-tiered mobile wireless sensor networks (TMWSNs).

In recent years, a novel paradigm of MEC, namely two-tiered mobile wireless sensor networks (TMWSNs) [8,9], has arisen and, because of the high robustness and scalability of its in-nature two-tiered network architecture, has attracted researchers’ attention [10,11]. As shown in Figure 1, a TMWSN model, which is different from traditional flat wireless sensor networks (WSNs) [12,13,14,15], consists of two layers [16]. The lower layer is composed of many resource-limited mobile (and static) sensor nodes responsible for monitoring the environment and sending reports to the upper layer, while the upper layer is made up of resource-rich storage nodes whose main responsibilities are to collect reports from the sensor nodes and respond to the sink. Clearly, the storage nodes are key in TMWSNs because of their roles in bridging sensor nodes and the sink. Thus, in a hostile environment, storage nodes are more prone to being captured and hacked, so the security of data transmission and processing through storage nodes in TMWSNs is under threat.

This paper focuses on the integrity preservation of spatial-temporal Top-*k* queries [17] in TMWSNs, where the selected top *k* data items should satisfy not only the time but also the geographic requirement [18] of a given Top-*k* query, and compares two typical schemes, namely VTMSN [9] and EVTopk [8]. The motivations of this paper are two-fold:

- It is a much more challenging problem to secure spatial-temporal Top-*k* queries in a mobile scenario than a static one in two-tiered wireless sensor networks, since malicious storage nodes may return false and/or incomplete Top-*k* query results to the sink with many more attacking options in a mobile scenario than a static one. For example, malicious storage nodes may replace sensing data generated by a mobile sensor node *S* when the data is in the queried region with data generated by the same node *S*, but not when the data is in the same region. As a result, although many schemes have been proposed to secure Top-*k* queries in static two-tiered wireless sensor networks, they are not fit for TMWSNs.
- The two typical schemes are directly correlated and deal with the same problem (the integrity preservation of spatial-temporal Top-*k* queries in TMWSNs) using different methods. Although the two schemes both declare that they are secure and efficient, they have never been compared with each other, and we never know which one performs much better or what shortcomings and advantages they have. In our opinion, it is very important to find out the exact answers to these questions so that we are able to improve these schemes.

In summary, our main contributions are as follows.

- We reveal the essence of the ideas in both VTMSN and EVTopk, and describe their fundamental principles on how to achieve integrity validation of query results of spatial-temporal Top-*k* queries in TMWSNs.
- We present our theoretical analysis about the performances of the two schemes on security and energy efficiency, and point out their advancements and shortcomings in theory.
- Through extensive simulations, we compare the two schemes. We find that the proof information in both schemes use a large part of the total information in the report packets sent from the sensor nodes to the storage nodes, as well as the information in the query results sent from the storage nodes to the sink. Specifically, the proportions of the proof information of both schemes in the report packets sent from the sensor nodes to the storage nodes are larger than 40%, and those in the query-result packets sent from the storage nodes to the sink are even higher than 80%.
- The reasons why so much proof information is included in the report packets and the query results are analyzed for both schemes, and some suggestions are given aiming to achieve a better scheme in the future research.

We organize the rest of this paper as follows. In Section 2, a summary of related work is presented; in Section 3, we analyze the theoretical essence of VTMSN and EVTopk; in Section 4, the security and the energy efficiency performances of both VTMSN and EVTopk are analyzed and compared in theory; in Section 5, results of extensive simulations are provided for comparison and analysis of the performances of the two schemes, especially in terms of their energy efficiency; in Section 6, the drawbacks of the two schemes are discussed, and some suggestions as well as plans for our future work are presented; finally, in Section 7, we conclude this paper.

## 2. Related Works

In recent years, many schemes have been proposed for preserving the integrity of Top-*k* queries in two-tiered wireless sensor networks. We can mainly classify them into the following categories: message-authentication-code-based (or MAC-based) schemes [19,20,21,22], data-items-binding-based schemes [23], data-aggregation-tree-based schemes [24,25], ID-broadcasting-based schemes [26], digital-watermark-based schemes [27], and dummy-readings-based schemes [28]. We present a brief summary of these schemes as follows.

The MAC-based schemes require each data item to be attached to a MAC as part of the proof information when it is sent to the corresponding storage node or the sink. Thus, the redundant data in the reports must be very large in such schemes, especially when there is a large quantity of data items generated in the cells. The main idea of data-items-binding-based schemes is to create correlations among data items generated by each sensor node in order to use binding technologies, such as encryption binding, so that adversaries cannot undermine the integrity of Top-*k* query results without secret keys. In the data-aggregation-tree-based schemes, a data aggregation tree, which takes the storage node as its root, is constructed in each cell. Each sensor node in the tree generates proof information for each data item that passes though it. The sink regenerates the proof information according to the topology structure of the tree to validate the integrity of the Top-*k* query results. In the ID-broadcasting-based schemes, each sensor node estimates whether it has the Top-*k* data items or not by comparing its own data items with a threshold value sent from the sink. If it has the Top-*k* data items, it will broadcast its ID across the cell. Any sensor node that receives the ID adds it to its own proof information with a certain probability. In this way, the sink can validate the integrity of Top-*k* query results with a certain success probability merely using part of the sensor nodes’ proof information in each cell. In digital-watermark-based schemes, a *t*-bits check code, which serves as a digital watermark, is generated for each data item and embedded into its neighboring data items. The sink validates the integrity of the Top-*k* query results exploiting the watermarks embedded in the data items. According to [27], if the length of the error bits is greater than t+l, the sink cannot ensure a 100% success probability to validate the integrity of the Top-*k* query results. The dummy-readings-based schemes achieve integrity validation of the Top-*k* query results by adding dummy readings to the normal sensing data items so that the malicious storage nodes cannot distinguish the normal ones from the dummy ones.

The common feature of the above-mentioned schemes is that they all focus on Top-*k* queries in static two-tiered sensor networks. They cannot ensure the security of spatial-temporal Top-*k* queries in TMWSNs because of the special attacking models, whereby data items generated in the queried region are replaced with those generated by the same sensor node/nodes but not in the same region, exploited by the adversaries. To the best of our knowledge, VTMSN [9] and EVTopk [8] are the only two schemes that have been proposed to achieve the integrity preservation of the spatial-temporal Top-*k* queries in TMWSNs in recent years. In the following, we will review the two schemes in greater detail and compare them thoroughly through computer security theory and extensive simulations.

## 3. Key Ideas of VTMSN and EVTopk

The common feature of VTMSN and EVTopk is that they all exploit methods of correlation and encryption to ensure the integrity of the spatial-temporal Top-*k* queries in TMWSNs. That is, they correlate data items generated by sensor nodes with their corresponding locations, generating time, and node IDs as well as their neighboring data items using encryption technologies. The encryption technology used by the two schemes is also the same, and the difference is in their correlation methods.

### 3.1. VTMSN

In VTMSN, each sensor node shares a different symmetric key with the sink. A sensor node $S_{i}$ correlates its own data items with itself, the corresponding generation location, and the time slot using encryption such as $E_{K_{i}}\left\{t||d||loc\right\}$ where $K_{i}$ is the symmetric key shared between the sensor node $S_{i}$ and the sink, *t* denotes the time slot when the data item was generated, loc is the location where the data item was generated, and *d* is the corresponding score of data item *D*. Data item *D* and its corresponding score *d* satisfy the equation d=f(D), where f(∗) is a public scoring function [29].

In order to decrease the quantity of proof information sent from the sensor nodes to the storage nodes, and those from the storage nodes to the sink, VTMSN distributes each location of any mobile sensor node in order, according to the sequence in which the sensor node moves, and binds the locations and their corresponding orders using encryption such as $E_{K_{i}}\left\{1|\right|{loc}_{1}\left||2|\right|{loc}_{2}\left||\ldots \right\}$, where ${loc}_{1}$ and ${loc}_{2}$ denotes the locations whose corresponding orders in the sequence are 1 and 2, respectively. Then, the data items only need to be bound with the orders of the locations rather than the locations themselves. Thus, the above-mentioned encryption item $E_{K_{i}}\left\{t||d||loc\right\}$ becomes $E_{K_{i}}\left\{t||d||j\right\}$ where *j* is the corresponding sequence order of the location loc. Because the length in bits of an order number is much shorter than that of the exact location data, this method can decrease the proof information, especially when there are many data items at each location.

To correlate the data items themselves, VTMSN first sorts the data items according to their scores and then binds the order number of each data item with its score using symmetric key encryption such as $E_{K_{i}}\left\{1|\right|d_{1}\},E_{K_{i}}\left\{2|\right|d_{2}\}$, and so on, where $d_{1}$ and $d_{2}$ denote the scores of the data items $D_{1}$ and $D_{2}$, respectively.

### 3.2. EVTopk

EVTopk binds the data items with their generation locations, time slots, and node IDs and correlates the data items themselves using the one-way hash chain.

Specifically, for any sensor node $S_{i}$ whose ID is *i*, it uses $HMAC_{K_{i,t}}\left\{i||t\right\}$, where $HMAC_{K_{i,t}}$ denotes a hash message authentication code keyed with $K_{i,t}$ to bind the node ID and the time slot. It then sorts the data items according to their corresponding data scores and uses a recursively computing method to correlate the neighboring data items, their generation location, time slot, and node ID. Suppose a sensor node $S_{i}$ had moved to $n_{i}$ locations and generated $n_{i}$ data items in total during time slot *t*. Let $D_{i,j}$ denote the $j^{th}$ data items generated by $S_{i}$ after the data items are sorted according to their corresponding scores, and let $v_{i,j}$ denote the proof information generated by $S_{i}$ for $D_{i,j}$. Then $v_{i,j}$ equals $HMAC_{K_{i,t}}\{v_{i,j+1}||loc_{i,j}\left|\right|d_{i,j}\}$, where $loc_{i,j}$ and $d_{i,j}$ are the generation location and the corresponding score of $D_{i,j}$, respectively, and $v_{i,n_{i}+1}$ equals $HMAC_{K_{i,t}}\left\{i||t\right\}$.

In this way, the sink only needs to recompute $v_{i,1}$ for each node $S_{i}$ using the materials in the spatial-temporal Top-*k* query result and the symmetric key shared between the sink and $S_{i}$. The sink compares the recomputed value of $v_{i,1}$ with that contained in the query result to validate the authenticity of the information included in the query result.

## 4. Security and Energy Efficiency Analysis

### 4.1. Security Analysis

The two schemes can all ensure that the sink is able to detect the incomplete spatial-temporal Top-*k* query results with a 100% probability in the case that the attacks are launched by the storage nodes only, the reasons for which are as follows.

To undermine the integrity of the Top-*k* query results, the attacking method used by the malicious storage node is replacement in nature, since the storage node should return *k* data items exactly to the sink in the condition that there are totally more than *k* data items generated by the queried sensor nodes in the queried region. Here, the word “replacement” refers to the following two options: (1) replace the qualified Top-*k* data items with forged ones; (2) replace the qualified Top-*k* data items with real but unqualified data items. If the malicious storage node takes the first option, both schemes can detect the incomplete Top-*k* query results with a 100% probability according to Theorem 1 in [8] and Theorem 6 in [9] as long as no sensor node is captured by the adversaries; if the storage node takes the second option, according to Theorem 1 in [8] and Theorem 9 in [9], both schemes are also able to detect the corresponding incomplete Top-*k* query results. It is notable here that the methods for the two schemes prevent the storage node from replacing the qualified Top-*k* data items with data items that are not generated in the queried region. The key idea of the methods used in the two schemes is to bind each data item with the location where it was generated using symmetric encryption [9] or a one-way hash chain [8]. By preventing the malicious storage node from undermining the binding relationship between each data item and its location, the two schemes are able to determine whether a data item is generated in the queried region or not.

Of course, the number of data items returned by the malicious storage node may be less than *k* even if the total number of data items generated by the queried sensor nodes in the queried region is no less than *k*, which means that the storage node implies that there is no more than *k* data items generated by all the queried sensor nodes in the queried region. In this case, some of the qualified Top-*k* data items must be dropped by the storage node. According to Theorem 9 in [9] and Theorem 2 in [8], the incomplete Top-*k* query results must be detected by the two schemes.

However, in the case that the sensor nodes can be captured and become malicious, the two schemes will not be secure anymore. In such a case, the compromised sensor nodes may launch collusion attacks with the storage nodes. As a result, both schemes cannot preserve the integrity of the spatial-temporal Top-*k* query results any longer even if only a small portion of the sensor nodes are captured because some of the symmetric keys shared between the sensor nodes and the sink are disclosed to the adversaries.

Another shortcoming of the two schemes is the privacy preservation of the data items generated by the sensor nodes. In TMWSNs, all sensing data items need to be sent to the storage nodes to be stored. To avoid being disclosed to the storage nodes, the data items must be sent to the storage nodes in ciphertext rather than plaintext. However, how can storage nodes determine the qualified Top-*k* data items in such a case? No solution is included in either scheme to solve this problem. In other words, neither of them can achieve privacy preservation for Top-*k* query processing in TMWSNs.

### 4.2. Energy Efficiency Analysis

The quantity of the proof information, which is included in both the reports of the sensor nodes and the query results supplied by the storage nodes, substantially affects the energy efficiency of the schemes. Generally speaking, the lower the amount of proof information included, the more energy efficiency a scheme can achieve. Thus, to analyze and compare the energy efficiency of VTMSN and EVTopk, we need to analyze the quantity difference of the proof information between VTMSN and EVTopk.

We first analyzed the difference in the proof information included in the reports of the sensor nodes between VTMSN and EVTopk. As assumed in [8], we assume each sensor node $S_{i}$ has moved to $n_{i}\left(n_{i}>0\right)$ locations in a time slot, and one data item is generated at each location. Then, according to [8], the proof information included in the report of any sensor node $S_{i}$ in each time slot in EVTopk contains one time slot number, one node ID, $n_{i}$ location items, and $n_{i}$ HMAC items. As for VTMSN, according to [9], the proof information included in each report of any sensor node $S_{i}$ contains $n_{i}+1$ time slot numbers, $n_{i}$ location items, $2n_{i}$ location IDs, and $n_{i}$ data score items. Through a comparison, we can see that all sensor reports in VTMSN, compared to those in EVTopk, contains $n_{i}$ more time slot numbers, $2n_{i}$ more location IDs, and $n_{i}$ more data scores, while reports in EVTopk, compared to those in VTMSN, includes one more node ID and $n_{i}$ more HMAC items.

We then analyzed the quantity difference of the proof information in the query results sent from the storage nodes to the sink between VTMSN and EVTopk. In both schemes, the storage nodes must add proof information generated by each sensor node to the query results. Specifically, in EVTopk, for sensor node $S_{i}$, the storage nodes should add the node ID *i*, $n_{i}$ location items and some other materials which can make the sink able to recalculate the item $v_{i,1}$ and compare the recalculated one with that included in the query result to see whether they are the same [8] or not. In VTMSN, $n_{i}$ location items and some other materials also need to be included in the query result for each sensor node $S_{i}$. The prominent differences between the proof information in the query result of EVTopk and that of VTMSN are the number of hash values and that of the data scores. In the proof information of EVTopk, there are at most two hash items and about $\sum_{i=0}^{|I_{t}|}\gamma_{i}-k$ data scores, where $|I_{t}|$ symbolizes the set of sensor nodes that moved into the queried region during the queried time slot, and $\gamma_{i}$ denotes the number of $S_{i}$’s data items whose scores are higher than the lowest score of the qualified Top-*k* data items. in the proof information of VTMSN, although there is no hash item, the maximum of the data scores can reach k+λ, where λ symbolizes the total number of locations that have been taken up by the sensor nodes in the queried region and time slot. Thus, the gap of the out-cell performances between VTMSN and EVTopk on energy efficiency mainly depends on the final exact numbers of data scores included in the proof information. In other words, if the final numbers of data scores included in the proof information of the two schemes are close to each other, or the data scores use only a small proportion of proof information in both schemes, the out-cell performances of the two schemes will be very close.

## 5. Performance Evaluation

In this section, we evaluate the energy efficiency of VTMSN and EVTopk through extensive simulations. The metrics we use are defined as follows:

- $C_{v\_sm}$: the extra communication cost of transmitting all proof information from the sensor nodes to the storage node in a cell in one epoch;
- $C_{v\_mw}$: the extra communication cost of transmitting proof information in a query result from the storage node to the sink;
- $R_{vs\_sm}$: the ratio between $C_{v\_sm}$ and the communication cost of transmitting all reports from the sensor nodes to the storage node in a cell in one epoch;
- $R_{vs\_mw}$: the ratio between $C_{v\_mw}$ and the communication cost of transmitting a query result from the storage node to the sink.

The simulator we use is OMNET++, and the default parameter settings, which are set by reference to [8,9,19] with slight changes, are listed in Table 1. As other default parameters can be interpreted well according to the description presented along with the parameters in Table 1, we explain the meaning of parameter $P_{m}$ further. Suppose the total number of sensor nodes in a cell is *N* and that of mobile ones is *n*. Then, we have

(1) $$P_{m}=n/N.$$

### 5.1. Performances on $C_{v\_sm}$ and $R_{vs\_sm}$

**Figure 2:**
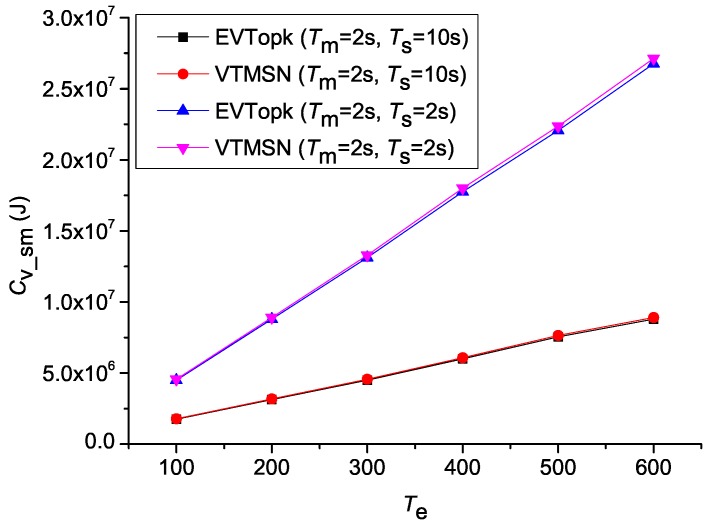
$C_{sm}$ with different settings of $T_{m}$, $T_{s}$, and $T_{e}$.

**Figure 3:**
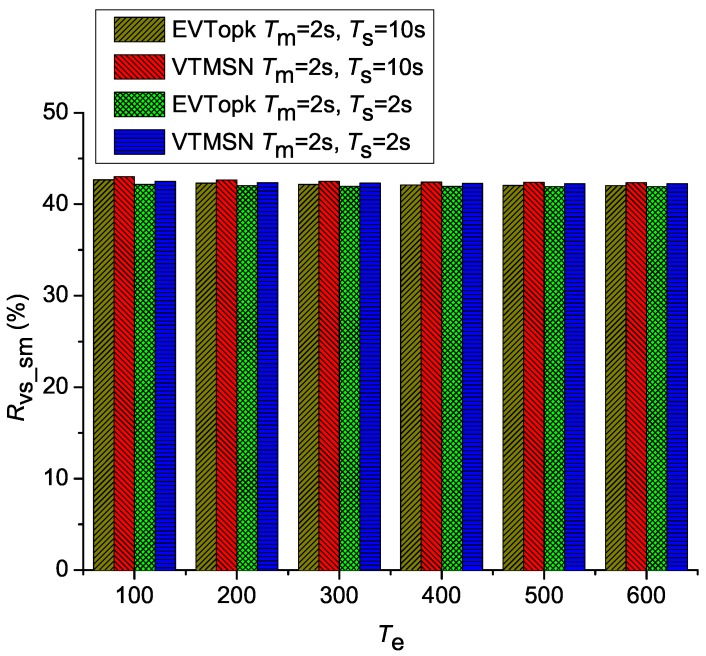
$R_{vs\_sm}$ with different settings of $T_{m}$, $T_{s}$, and $T_{e}$.

**Figure 4:**
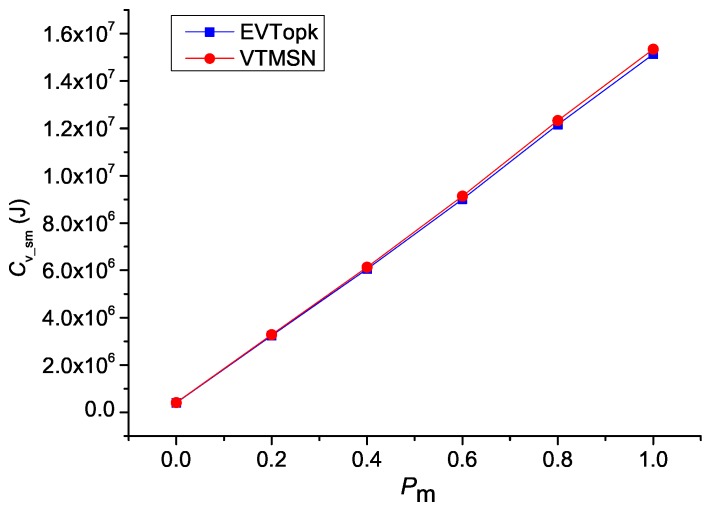
$C_{sm}$ with different settings of $P_{m}$.

**Figure 5:**
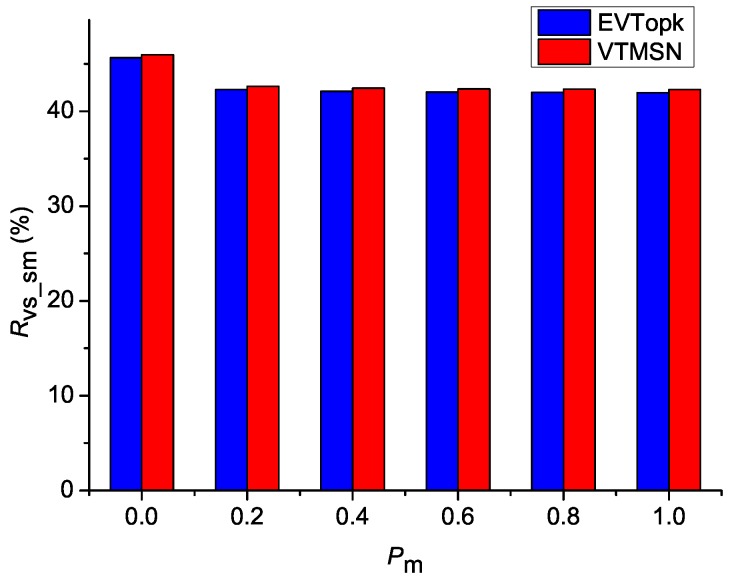
$R_{vs\_sm}$ with different settings of $P_{m}$.

Figure 2 and Figure 3 illustrate the simulation results of $C_{v\_sm}$ and $R_{vs\_sm}$ in both EVTopk and VTMSN with different settings of $T_{s}$, $T_{m}$, and $T_{e}$, and Figure 4 and Figure 5 show the results of $C_{v\_sm}$ and $R_{vs\_sm}$ with different settings of $P_{m}$. From those results, we find that the two schemes perform similarly in energy efficiency. Moreover, the energy-consumption proportions of transmitting the proof information account for more than 40% of the total communication cost between the sensor nodes and the storage nodes in both schemes.

### 5.2. Performances on $C_{v\_mw}$ and $R_{vs\_mw}$

**Figure 6:**
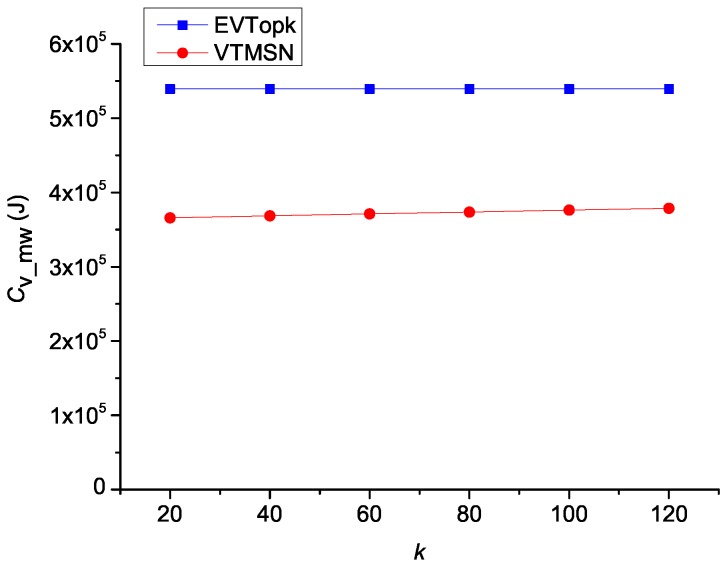
$C_{mw}$ with different settings of *k*.

**Figure 7:**
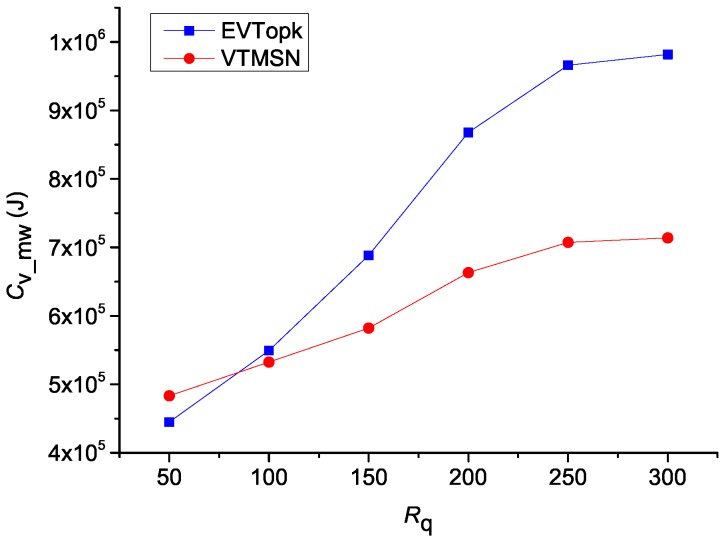
$C_{mw}$ with different settings of $R_{q}$.

**Figure 8:**
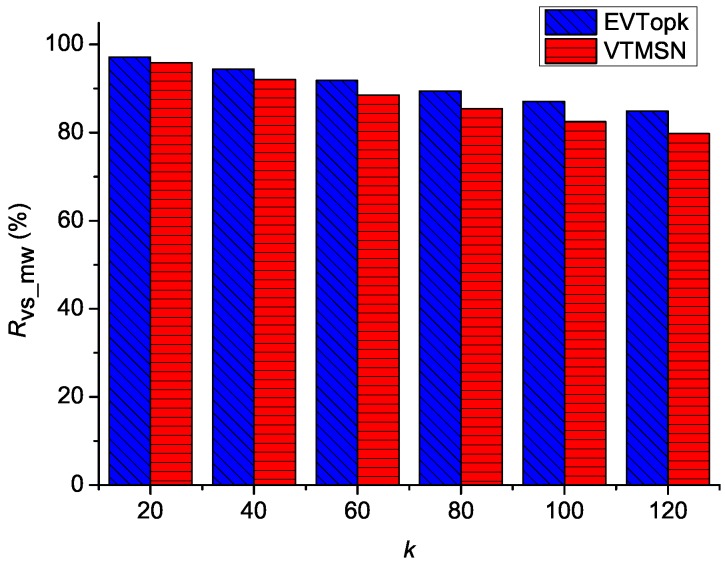
$R_{vs\_mw}$ with different settings of *k*.

**Figure 9:**
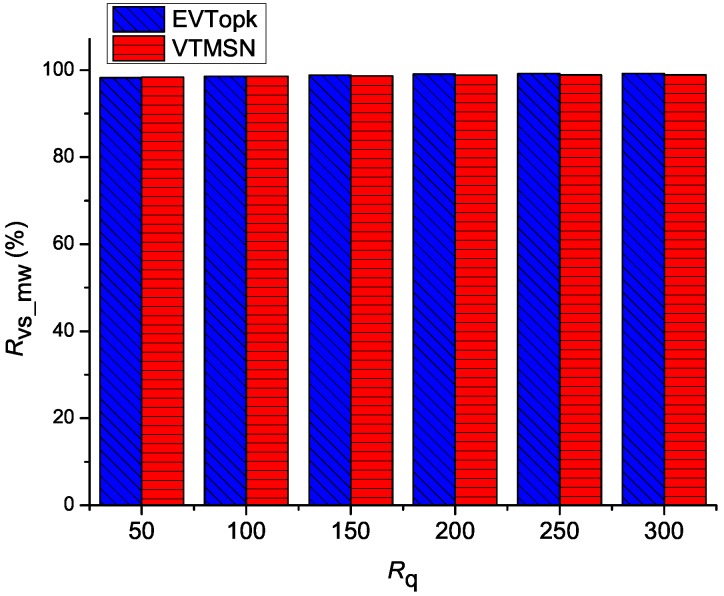
$R_{vs\_mw}$ with different settings of $R_{q}$.

The simulation results of $C_{v\_mw}$ in both EVTopk and VTMSN with different settings of *k* and $R_{q}$ are shown in Figure 6 and Figure 7, and the corresponding results of $R_{vs\_sm}$ are illustrated in Figure 8 and Figure 9 respectively. From those figures, we can see that, in most cases, VTMSN achieves lower $C_{v\_mw}$ and $R_{vs\_sm}$. However, the energy efficiency of VTMSN is not high, and the proportion of energy consumption on transmitting the proof information in VTMSN accounts for more than 80% of the total communication cost between the storage nodes and the sink.

## 6. Discussion

From the simulation results, we can see that one of the common disadvantages of the two schemes is that the extra communication costs, which are brought about by transmitting the proof information not only between the sensor nodes and the storage nodes but also between the storage nodes and the sink, are too high. Generally speaking, the proportion of the extra communication cost in the total communication cost should be less than 10% rather than more than 40% or even 80% in the two schemes.

The communication cost of the two schemes is high mainly because of the large amount of location data in the proof information. To make the sink know where exactly each data item is generated in TMWSNs, the two schemes encrypt the generation location of each data item and take it as part of the proof information. In a 2D coordinate system, a location tuple consists of two elements: the abscissa data and the ordinate data. Thus, if each element occupies 64 bits of computation space, a location tuple can totally occupy 128 bits. According to the default parameter settings of the two schemes, the bit length of a data item is 400 bits. Based on the numeric comparison, why the location information leads to a high extra communication cost in the two schemes becomes clear.

Thus, to achieve a more efficient and safer scheme in securing spatial-temporal Top-*k* queries in TMWSNs, our suggestions are listed as follows:

(1) The location information in both the reports sent from the sensor nodes to the storage nodes and the query results sent from the storage nodes to the sink should be reduced as much as possible on the condition that the sink can validate the integrity of the Top-*k* query results using the proof information included in the query results.
(2) Novel techniques of data association encryption, such as the blockchain-based technique [30], can be used to further improve the efficiency and the integrity preservation of Top-*k* query processing in TMWSNs. Blockchain-based security technology has been developing substantially in recent years, and it is being used to design schemes that meet the special security requirements of different Internet of Things applications, including the securing of Top-*k* query processing in TMWSNs, because of the technology’s high temper resistance and efficiency.
(3) Privacy preservation for Top-*k* query processing in TMWSNs should be achieved using the order-preserving encryption schemes (e.g., OPESs (order-preserving encryption schemes) [31] and MOPE (modular order-preserving encryption) [32]), along with pairwise key encryption schemes (e.g., the schemes proposed in [33,34]). The ciphertexts produced by using the OPESs can preserve the natural ordering of plaintexts if such plaintexts are one-dimensional data items, which is fit for Top-*k* query processing without decrypting the ciphertexts. Since the sensed data items generated by the sensor nodes may be multidimensional, pairwise key encryption schemes should also be adopted where keys are only shared by the sensor nodes and the sink.
(4) The scenario that the sensor nodes are asleep and awake dynamically [35] should be considered when solving the above-mentioned security problem of spatial-temporal Top-*k* queries in TMWSNs. The two typical schemes studied in this paper mainly consider the case where sensor nodes are always awake and continue working during their whole lifetime. In this case, all sensor nodes are assumed to report their collected data to their corresponding storage node/nodes at the end of each epoch. However, in the duty-cycled scenario [35], some sensor nodes that are asleep may fail to report their collected data at the end of each epoch, which could be a point of penetration for malicious storage nodes to undermine the integrity of the Top-*k* query results. For example, malicious storage nodes may drop all of the collected data of awake sensor nodes and claim that they are asleep.

## 7. Conclusions

This paper compares two typical schemes, namely EVTopk and VTMSN, proposed for securing spatial-temporal Top-*k* queries in the novel MEC paradigm TMWSNs. Through theory analysis, we show that the two schemes can achieve the integrity validation of the spatial-temporal Top-*k* query results in TMWSNs with little difference on the proof-information quantity in both the reports generated by the sensor nodes and the query results returned from the storage nodes to the sink. Extensive simulation results also show that the two schemes have similar energy efficiency. The common disadvantage of the two schemes is that the extra communication cost brought by transmitting the proof information is too high. Specifically, the ratio of the extra communication cost to the total communication cost between the sensor nodes and the storage nodes is larger than 40% and, between the storage nodes and the sink, 80%. Through a deep analysis, we find that the main reason that leads to this weakness is the large quantity of location data included in the proof information.

With regard to our future work, we plan to achieve the ideas suggested in Section 6 and come up with solutions that are much more preferable and have greater energy efficiency and better security performance for spatial-temporal Top-*k* query processing in TMWSNs, using technologies such as geographic gridding, location virtualization, and blockchain-based technologies.

## Figures and Tables

**Table 1:** Default parameter settings.

Parameters	Default Values
$P_{m}$ (Proportion of the mobile sensor nodes among all the sensor nodes)	0.5
$T_{e}$ (Time length of one epoch)	100 s
$T_{s}$ (Time length to keep static for a mobile sensor node at any target location)	10 s
$T_{m}$ (Time length to keep moving for a mobile sensor node)	2 s
*N* (Total number of sensor nodes in a cell)	300
$C_{size}$ (Size of a cell)	400 × 400 m${}^{2}$
*R* (Communication radius of any sensor node)	100 m
$R_{qr}$ (Radius of the queried region in a cell)	200 m
$l_{t}$ (Bit length of the value of a queried time point)	32 bits
$l_{ID}$ (Bit length of an ID number)	16 bits
$l_{D}$ (Bit length of a sensing data item)	400 bits
$l_{score}$ (Bit length of the score of a sensing data item)	20 bits
$l_{order}$ (Bit length of an order number)	16 bits
$l_{hash}$ (Bit length of a Hash value)	160 bits
$l_{loc}$ (Bit length of each location tuple)	128 bits
